# Use of Recombinant Virus Replicon Particles for Vaccination against *Mycobacterium ulcerans* Disease

**DOI:** 10.1371/journal.pntd.0004011

**Published:** 2015-08-14

**Authors:** Miriam Bolz, Sarah Kerber, Gert Zimmer, Gerd Pluschke

**Affiliations:** 1 Swiss Tropical and Public Health Institute, Basel, Switzerland; 2 University of Basel, Basel, Switzerland; 3 Institute of Virology and Immunology (IVI), Mittelhäusern, Switzerland; Fondation Raoul Follereau, FRANCE

## Abstract

Buruli ulcer, caused by infection with *Mycobacterium ulcerans*, is a necrotizing disease of the skin and subcutaneous tissue, which is most prevalent in rural regions of West African countries. The majority of clinical presentations seen in patients are ulcers on limbs that can be treated by eight weeks of antibiotic therapy. Nevertheless, scarring and permanent disabilities occur frequently and Buruli ulcer still causes high morbidity. A vaccine against the disease is so far not available but would be of great benefit if used for prophylaxis as well as therapy. In the present study, vesicular stomatitis virus-based RNA replicon particles encoding the *M*. *ulcerans* proteins MUL2232 and MUL3720 were generated and the expression of the recombinant antigens characterized *in vitro*. Immunisation of mice with the recombinant replicon particles elicited antibodies that reacted with the endogenous antigens of *M*. *ulcerans* cells. A prime-boost immunization regimen with MUL2232-recombinant replicon particles and recombinant MUL2232 protein induced a strong immune response but only slightly reduced bacterial multiplication in a mouse model of *M*. *ulcerans* infection. We conclude that a monovalent vaccine based on the MUL2232 antigen will probably not sufficiently control *M*. *ulcerans* infection in humans.

## Author Summary

Infection with *Mycobacterium ulcerans* can lead to a slow progressing, ulcerative disease of the skin and underlying soft tissue called Buruli ulcer. The disease is most prevalent in rural African communities with limited access to health care facilities. The most efficient means to prevent the disease, a vaccine against Buruli ulcer is not available to date. In the present study we investigated the immunogenicity and protective potential of a single cycle virus system expressing the two *M*. *ulcerans* antigens MUL2232 and MUL3720. Immunization of mice with those vesicular stomatitis virus replicon particles led to the induction of humoral as well as cellular immune responses in the immunized animals. Subsequent challenge experiments in a mouse model of *M*. *ulcerans* infection demonstrated only a limited reduction of bacterial burden in mice immunized with a prime-boost approach with MUL2232. Most probably, a vaccine formulation with only one antigen will not be able to provide protection against Buruli ulcer in humans.

## Introduction

*Mycobacterium ulcerans* causes Buruli ulcer (BU), a disease of the skin and underlying subcutaneous tissue, which is reported from over 30 countries worldwide [1]. BU is most prevalent in West African countries and mainly affects children less than 15 years of age, living in remote, rural areas [2]. The natural reservoir of *M*. *ulcerans* has not been identified so far and also the mode of transmission of this pathogen remains unclear [3].

*M*. *ulcerans* produces a macrolide exotoxin called mycolactone, which induces apoptosis in mammalian cells and leads to the typical clinical presentation of ulcerative BU skin lesions after the overlying epidermis has collapsed [4]. Non-ulcerative forms of the disease are nodules or papules, oedema and plaques [5]. BU was traditionally treated by wide surgical excision of the affected skin and skin grafting if necessary. Since 2004, treatment of patients for eight weeks with the antibiotics rifampicin and streptomycin is recommended as standard therapy by the World Health Organization (WHO) [6]. Even though the use of antibiotics has reduced recurrence rates to less than 2% [7–9], patients are often left with scars and lifelong disabilities [10]. A vaccine against BU would therefore be of high value to prevent and treat the disease [11].

As opposed to *M*. *tuberculosis* and *M*. *leprae*, which are both intracellular pathogens for which T helper 1 (T_H_1) cellular immune responses are essential for infection control [12], *M*. *ulcerans* is found in extracellular clumps in the necrotic subcutaneous skin tissue of advanced lesions. However, there is evidence for an initial intracellular stage in macrophages during the early phase of infection [13,14]. Correlates of protection have not been identified, and in particular it is not known, whether antibodies specific for surface antigens of *M*. *ulcerans* have protective activity [15].

Vaccination with Bacille Calmette-Guérin (BCG) seems to confer a transient protection from BU and a shorter duration of ulcers [16–18]. In a mouse model of *M*. *ulcerans* infection, vaccination with either BCG or a mycolactone-negative *M*. *ulcerans* mutant strain delayed the onset of foot pad swelling [19,20]. A limited protective efficacy has also been achieved with monovalent DNA-based protein subunit vaccine formulations targeting either the mycolyl-transferase antigen (Ag) 85A from *M*. *bovis* or *M*. *ulcerans* [21,22] or mycolactone polyketide synthase domains that are encoded on the giant *M*. *ulcerans* plasmid pMUM001 [23].

Vesicular stomatitis virus (VSV) is a member of the virus family *Rhabdoviridae* and has a non-segmented single-stranded RNA genome of negative polarity. VSV is transmitted by insects and causes a vesicular-like disease in livestock and Flu-like symptoms in humans. The VSV seroprevalence is very low in the human population, indicating that human infections are rare. The non-persisting replication of VSV in the cytosol of the host cell, low virulence and low pre-existing immunity in humans, and the option of simple genetic manipulation [24] has recommended VSV as viral vector system for vaccination [25,26]. Several VSV-based vaccines for protection against viral and bacterial pathogens have been generated in recent years and have been evaluated in a number of animal models [27–30]. More recently, single-cycle VSV vectors have been developed, which lack the genetic information for the VSV glycoprotein G and are complemented with this protein *in trans*. Although these VSVΔG vectors are restricted to a single round of infection, they were shown to be as immunogenic as propagation-competent VSV vectors [31].

In the present study we used this safe vaccine vector platform to characterize the immunogenicity of two *M*. *ulcerans* protein antigens. In view of the mainly extracellular nature of *M*. *ulcerans*, we chose antigens known to be highly expressed on the surface of the bacteria. The 18 kDa small heat shock protein (MUL2232) was identified amongst the most immunodominant antigens expressed by *M*. *ulcerans* when serum responses from people living in endemic areas were analysed [32]. A homologue of MUL2232 was found in the genome of *M*. *leprae* but neither in *M*. *bovis* nor in *M*. *tuberculosis*. Similar to the *M*. *leprae* homologue, MUL2232 is associated with the cell wall fraction of *M*. *ulcerans* [32,33]. The second antigen chosen, MUL3720, is a 21 kDa protein with a putative lectin and a peptidoglycoan-binding domain that does not have homologues in *M*. *leprae* or *M*. *tuberculosis* [34,35]. It is highly expressed by *M*. *ulcerans* and may play a role in cell attachment and cell-cell interactions [35], making it a candidate antigen for vaccine development. We generated recombinant single-cycle VSV vectors encoding the selected antigens individually and analysed their expression in mammalian cell lines. We further analysed the immunogenicity of the generated VSV vectors and evaluated two short immunization protocols. Finally, we investigated the protective potential of such immunization schemes in an experimental mouse model of *M*. *ulcerans* infection.

## Materials and Methods

### Ethics statement

All animal experiments were conducted in compliance with the Swiss animal protection law and approved by the animal welfare committee of the Canton of Basel (authorization number 2375) and the Canton of Vaud (authorization number 2657).

### Cells

Baby hamster kidney 21 (BHK-21) cells were obtained from the German Cell Culture Collection (DSZM, Braunschweig). Cells were grown in Earle’s minimal essential medium (MEM, Life Technologies) supplemented with 5% foetal bovine serum (FBS, Biowest). BHK-G43, a transgenic BHK-21 cell line expressing the VSV G protein in an inducible manner, was maintained as described previously [36]. Murine L929 fibroblasts (ATCC, The Global Bioresource Center) were cultivated in RPMI medium (Gibco) supplemented with 10% foetal calf serum (FCS, Sigma), 2 mM glutamine (Gibco) and 0.05 mM β-mercaptoethanol (Gibco).

### Generation of recombinant vesicular stomatitis virus replicon particles expressing *M*. *ulcerans* codon optimized antigens

The potential protein vaccine candidate antigens MUL_2232 (GenBank accession number 4550596) and MUL_3720 (GenBank accession number 4553013) of *M*. *ulcerans* Agy99 were ordered as codon optimized genes for expression in humans (GenScript) and received in pUC57 plasmids. For generation of recombinant VSV replicon particles (VRPs), codon optimized target antigens were amplified by PCR and inserted into the pVSV*ΔG plasmid using single MluI and BstEII restriction sites upstream and downstream of the fourth transcription unit, replacing the VSV G gene [37]. Sequence integrity of the resulting plasmids was confirmed by Sanger sequencing. Recombinant VRPs were generated as described previously [38]. In brief, BHK-G43 cells were infected with recombinant MVA-T7 virus expressing T7 RNA polymerase [39]. Subsequently, the infected cells were transfected with plasmids driving T7 RNA polymerase-mediated expression of the VSV proteins N, P, and L, and with pVSV*ΔG(MUL2232) or pVSV*ΔG(MUL3720) driving T7 RNA polymerase-mediated transcription of VSV antigenomic (negative-sense) RNA. Expression of the VSV G protein was induced by adding mifepristone (Sigma) to the cell culture medium. At 24 hours post transfection cells were detached with trypsin and seeded along with an equal number of fresh BHK-G43 cells into T-75 flasks. Cells were then incubated at 37°C for another 24 hours in the presence of mifepristone. Cell culture supernatant was clarified by low-speed centrifugation and by passage through a 0.2 μm pore filter. The VRPs in the clarified cell culture supernatant were further propagated on mifepristone-induced BHK-G43 cells and stored at -70°C. VRPs were titrated on BHK-21 cells taking advantage of the eGFP reporter protein.

### Western blot analysis

#### Detection of vaccine antigens in VRP-infected cells

Equal numbers of confluent L929 fibroblasts were infected with VRPs at a multiplicity of infection (MOI) of 10 for 6 hours at 37°C. Cells were washed with ice-cold phosphate buffered saline (PBS; Sigma) and harvested for lysis. Protein lysates of both the soluble and insoluble fraction of the cells were produced with a cell fractionation kit (Abcam) according to the manufacturer’s instructions. Equal amounts of protein lysates as well as *M*. *ulcerans* lysate in an appropriate dilution were resolved on prefabricated 4–12% gradient gels (NuPAGE Novex 4–12% Bis-Tris Gel; Invitrogen) with MES running buffer (Invitrogen) according to the manufacturer’s directions. A dry-blotting system (iBlot; Invitrogen) was used to transfer proteins to nitrocellulose membranes. Membranes were blocked and specific proteins detected with in house anti-MUL2232 or anti-MUL3720 mouse mAbs followed by HRP-conjugated goat anti-mouse IgG γ-chain mAb (Southern Biotech, 1030–05). Blots were developed using ECL Western blotting detection reagents (ECL Western blotting Substrate; Pierce).

#### Cross-reactivity of the elicited antibodies in mouse sera with the native antigen in *M*. *ulcerans* lysate

10 μg of *M*. *ulcerans* whole cell lysate was resolved on a one well prefabricated 4–12% gradient gel (NuPAGE Novex 4–12% Bis-Tris Gel; Invitrogen) with MES running buffer according to the manufacturer’s instructions. The blocked membrane was subsequently cut into thin strips that were individually incubated with appropriate dilutions of serum of immunized mice for 2 hours. Secondary antibody and development were performed as described above.

### Immunofluorescence analyses

For indirect immunofluorescence analysis, BHK-21 cells were grown on 12 mm diameter cover slips (2 x 10^5^ cells/well) and infected with VRPs (10^6^ infectious units/well) for 6 hours at 37°C. Cells were fixed with 3% paraformaldehyde (PFA) and washed with PBS containing 0.1 M of glycine. The cells were permeabilized with 0.25% (v/v) Triton X-100 and subsequently incubated for 1 hour with anti-MUL2232 or anti-MUL3720 mAbs in appropriate dilutions in 1% bovine serum albumin (Sigma). For detection of antigen-bound primary antibodies, cells were incubated with an Alexa Flour 546 labelled anti-mouse IgG secondary antibody (1/500; Molecular Probes, A-11018). The cells were washed with distilled water, embedded in Mowiol 4–88 (Sigma) mounting medium, and analyzed with a Leica TCS SL confocal microscope and LCS software (Leica Microsystems AG, Glattbrugg, Switzerland).

### ELISA on recombinant protein

ELISA plates (Maxisorp; Nunc) were coated with 10ug/ml purified recombinant MUL2232 (rMUL2232) or MUL3720 (rMUL3720) protein produced in *Escherichia coli* [40]. After blocking, plates were incubated with serially diluted sera from immunized mice. Alkaline phosphatase-conjugated goat anti-mouse antibody (Sigma) was used as secondary antibody and *p*-nitrophenyl phosphate (Sigma) served as substrate. The optical density (OD) of the reaction product was measured at 405 nm with a microplate reader (Sunrise Absorbance Reader; Tecan). The threshold for endpoint titer determination was defined as the double of the mean measurements plus the mean standard deviation of a dilution series done without primary antibody and a dilution series done with pre-bleed serum. Individual serum dilution series were approximated with sigmoidal dose-response curves and the reciprocal dilution of the intersection between the curve and the threshold was defined as individual endpoint titer.

### Mouse immunization studies

All animal studies were conducted in 8 weeks old female BALB/c mice (Janvier). VRPs (10^7^ fluorescence-forming units) were either applied subcutanously (s.c.) in the neck (for evaluation of the vaccination protocol) or intramuscularly (i.m.) into the right caudal tight muscle using a volume of 100 μl or 30 μl, respectively. When several immunizations were conducted, injections were performed in three week intervals. For prime-boost vaccination regimen, 30 μg of non-adjuvanted recombinant protein were applied s.c. into the neck. Blood was collected from the tail vein prior to every immunization as well as 5/6 and 14 days after the protein boost. Serum was prepared by centrifugation of the blood in SST Microtainer tubes (Becton, Dickinson and Company).

### Analysis of cellular immune responses

Immunized mice were euthanized 3 weeks after the second immunization. Heart blood was collected and spleens were aseptically removed and homogenized by passing through a 70 μm cell strainer (BD Falcon). Cells were then pelleted and red blood cells lysed by incubating the pellet in red blood cell lysing buffer (Sigma) for 1 minute. Remaining cells were washed several times with stemline T-cell expansion medium (Sigma) supplemented with 4mM L-Glutamine (Gibco), 1% Pen-Strep (Gibco), 2.5 ug/ml Amphotericin B (Sigma) and 0.05 mM β-mercaptoethanol (Gibco) and finally adjusted to 4 x 10^6^ white blood cells/ml. 7.2 x 10^5^ cells per well were incubated in round-bottom microwell plates (BD Falcon) in a humidified CO_2_ incubator and stimulated with Concanavalin A (2 μg/ml, Sigma) as positive control or recombinant proteins at 5 μg/ml. Supernatants were harvested after 24h for Interleukin 2 (IL-2) and 96h for Interleukin 10 (IL-10) and Interferon gamma (IFNγ) assays and stored frozen at -20°C until analysis for the selected cytokines. Amount of cytokines in supernatants was determined with Quantikine ELISA kits for IL-2, IL-10 and IFNγ (R&D Systems).

### Challenge infection experiments

The *M*. *ulcerans* strain (S1013) used for the experimental infection of mice was isolated in 2010 from the ulcerative lesion of a Cameroonian BU patient [2]. Bacteria were cultivated for 6 weeks in Bac/T medium (Biomerieux), recovered by centrifugation, and suspended in sterile PBS to 125 mg/ml wet weight corresponding to 2.8 x 10^5^ CFU/ml as determined by plating serial dilutions on 7H9 agar plates (Difco). Three weeks after the last immunization, mice were infected with 30 μl of *M*. *ulcerans* suspension (1/100 of the stock solution in PBS) into the left hind foot pad. Development of the infection was followed by weekly measurements of the foot pad thickness with a caliper. At day 60 after experimental infection, mice were sacrificed and foot pads aseptically removed for enumeration of *M*. *ulcerans* bacteria or histopathology. Draining inguinal lymph nodes of designated animals were removed and fixed in formalin as well. All *M*. *ulcerans* infection experiments were conducted under BSL3 conditions.

### Colony forming unit (CFU) plating and enumeration of bacterial load by real-time PCR (qPCR)

Mouse feet designated for enumeration of *M*. *ulcerans* bacteria were immediately removed above the ankle after euthanasia, shortly dipped into 70% ethanol, then dried under the laminar flow, cut in 4 pieces with a scalpel and transferred to 750 μl of Bac/T medium in reinforced hard tissue grinding tubes (MK28-R, Precellys). Tissue homogenization was performed with a Precellys 24-Dual tissue homogenizer (3 x 20 s at 5000 rpm with 30 s break), the lysate was transferred to a new tube and the lysis tube still containing tissue remains was refilled with 750 μl of Bac/T medium. The remains were homogenized a second time and the two lysates pooled.

#### CFU plating

250 μl of the foot pad lysate was decontaminated with 0.5 M NaOH as described previously [41]. The pellet of decontaminated lysate was dissolved in 500 μl of Bac/T medium and appropriate dilution series plated on 7H9 agar plates. Following 150 days of incubation at 30°C colonies were counted and the concentration of bacteria expressed as CFU.

#### DNA extraction and qPCR

DNA from 100 μl of a 1 to 50 dilution of the foot pad lysate in PBS was extracted as described by Lavender and Fyfe [42]. Extracted DNA was analysed for IS2404 by qPCR as previously described [42]. Ct values were converted into genome copy numbers per foot pad by applying the standard curve established for IS2040 by *Fyfe et al*. [43].

### Histopathology

Mouse feet designated for histopathological analysis were removed above the ankle and immediately transferred to 10% neutral-buffered Formalin solution (approx. 4% formaldehyde, Sigma) for fixation during 24 hours at room temperature. Subsequently, the feet were decalcified in 0.6 M EDTA and 0.25 M citric acid for 12 days at 37°C and transferred to 70% ethanol for storage and transport. The samples were dehydrated and embedded into paraffin. 5 μm thin sections were cut, deparaffinised, rehydrated, and stained with Haematoxylin/Eosin (HE, Sigma, J.T. Baker) or Ziehl-Neelsen/Methylene blue (ZN, Sigma) according to WHO standard protocols [44]. Stained sections were mounted with Eukitt mounting medium (Fluka). Pictures were taken with a Leica DM2500B microscope or with an Aperio scanner.

### Statistical analysis and image processing

Differences of bacterial load in infected foot pads were statistically analysed by the Mann-Whitney test using Graph Pad Prism (Version 6.03). Results of cytokine production were subjected to log_10_ transformation and subsequently analysed with SAS software (SAS Institute, Cary, USA, release 9.3) using linear mixed models adjusted for random effects. Image processing and picture panel assembly was performed with Photoshop software (Adobe Photoshop CS6 Extended, version 13.0.1).

## Results

### Expression of the *M*. *ulcerans* vaccine candidate antigens MUL2232 and MUL3720 by cells infected with recombinant virus replicon particles

**Fig 1:**
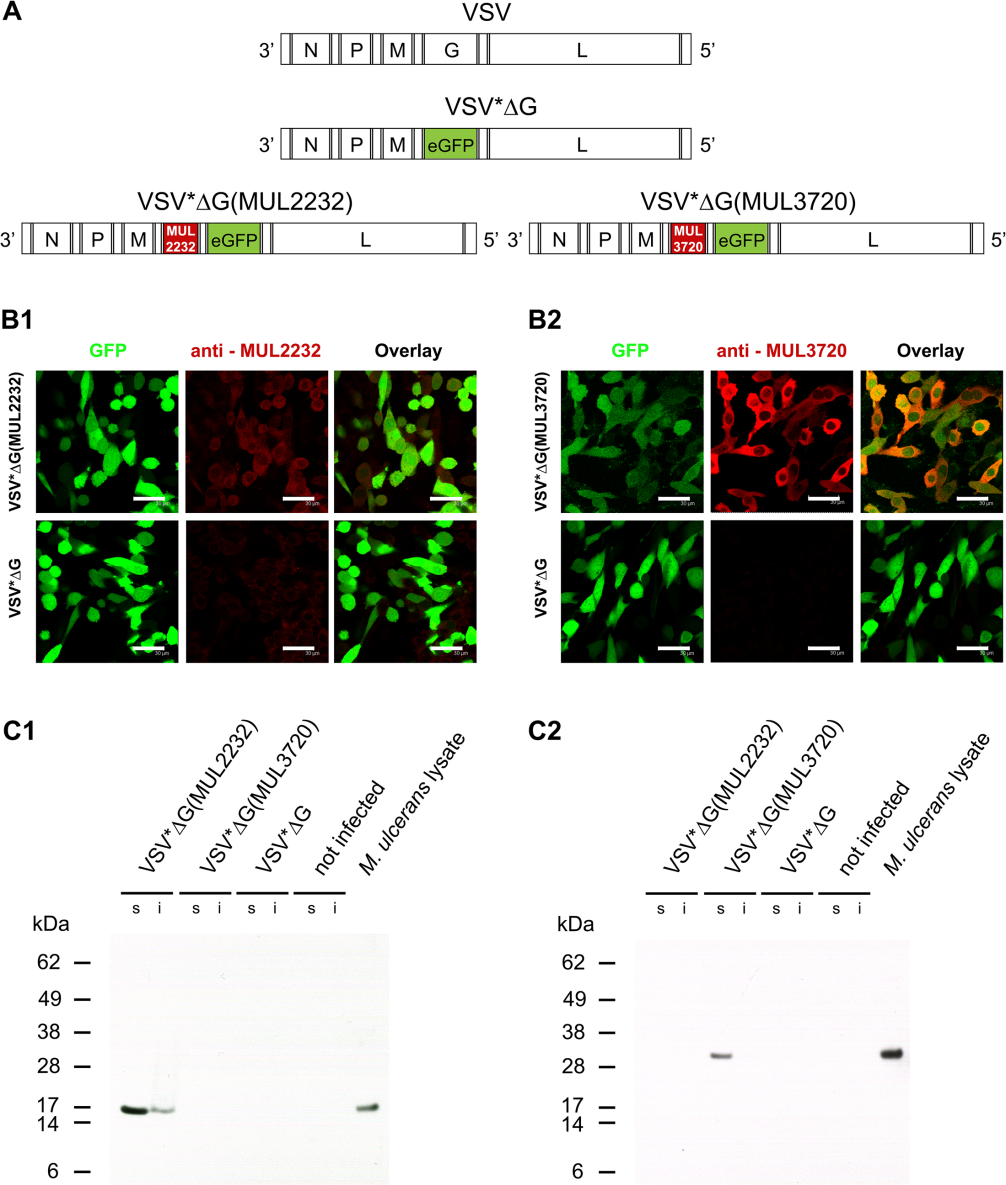
Expression of *M*. *ulcerans* proteins by VRP-infected cells. (A) Genome maps of recombinant VSV: The genome of VSV encodes for the nucleoprotein N, phosphoprotein P, matrix protein M, glycoprotein G, and the large RNA polymerase L. In VSV*ΔG, the glycoprotein G coding region was replaced by the coding sequence for eGFP. In VSV*ΔG(MUL22332) and VSV*ΔG(MUL3720), the VSV G gene was replaced by the *M*. *ulcerans* genes MUL2232 and MUL3720, respectively. The reporter protein eGFP is encoded by an additional transcription cassette located downstream of the *M*. *ulcerans* genes. (B) BHK-21 cells were infected with the indicated replicons and indirect immunofluorescence analysis performed with mAbs specific for MUL2232 (B1) or MUL3720 (B2) (red fluorescence). The infection of cells with VRPs is indicated by eGFP expression (green fluorescence). Scale bar equals 30 μm. (C) Equal numbers of L929 fibroblasts were infected with the indicated VRPs at a MOI of 10. At 6 hours post infection, the cells were harvested and protein lysates of the soluble (s) and insoluble (i) fraction analysed by Western blotting for expression of *M*. *ulcerans* antigens using mAbs specific for MUL2232 (C1) and MUL3720 (C2), respectively.

Candidate vaccines were generated by replacing the VSV glycoprotein G gene with the *M*. *ulcerans* genes MUL2232 or MUL3720 (Fig 1A). In order to ease virus detection and titration, the coding sequence for the enhanced green fluorescent protein (eGFP) was inserted as an additional transcription cassette downstream of the *M*. *ulcerans* genes. The recombinant viruses VSV*ΔG(MUL2232) and VSV*ΔG(MUL3720) as well as the control virus VSV*ΔG, which only contained the eGFP gene in place of the VSV surface glycoprotein G (Fig 1A), were produced and propagated in the helper cell line BHK-G43 providing the VSV G protein *in trans*. As expected from other studies with similar constructs, the trans-complemented particles were able to infect a variety of different mammalian cell lines but were unable to release progeny viruses [27,45]. We thus refer to them as to virus replicon particles (VRPs).

In order to study the expression of MUL2232 and MUL3720 in infected cells, BHK-21 cells were infected with the three different VRPs generated and analysed by using MUL2232 and MUL3720-specific mouse monoclonal antibodies (mAbs) in immunofluorescence microscopy. Both antigens accumulated in the cytosol of the infected BHK-21 cells (Fig 1B1 and 1B2). While in the immunofluorescence analysis MUL2232 appeared to be expressed at lower levels than MUL3720, staining intensities in Western blotting analyses were comparable for both proteins (Fig 1C). MUL2232 was mainly detected in the soluble fraction of infected L929 fibroblasts and to a smaller part in the insoluble fraction (Fig 1C1) while MUL3720 was found in the soluble fraction only (Fig 1C2). Both proteins co-migrated with the corresponding proteins in *M*. *ulcerans* lysate according to the predicted molecular mass (Fig 1C).

### Immunization of mice with virus replicon particles induces *M*. *ulcerans* cross-reactive antibody responses

**Fig 2:**
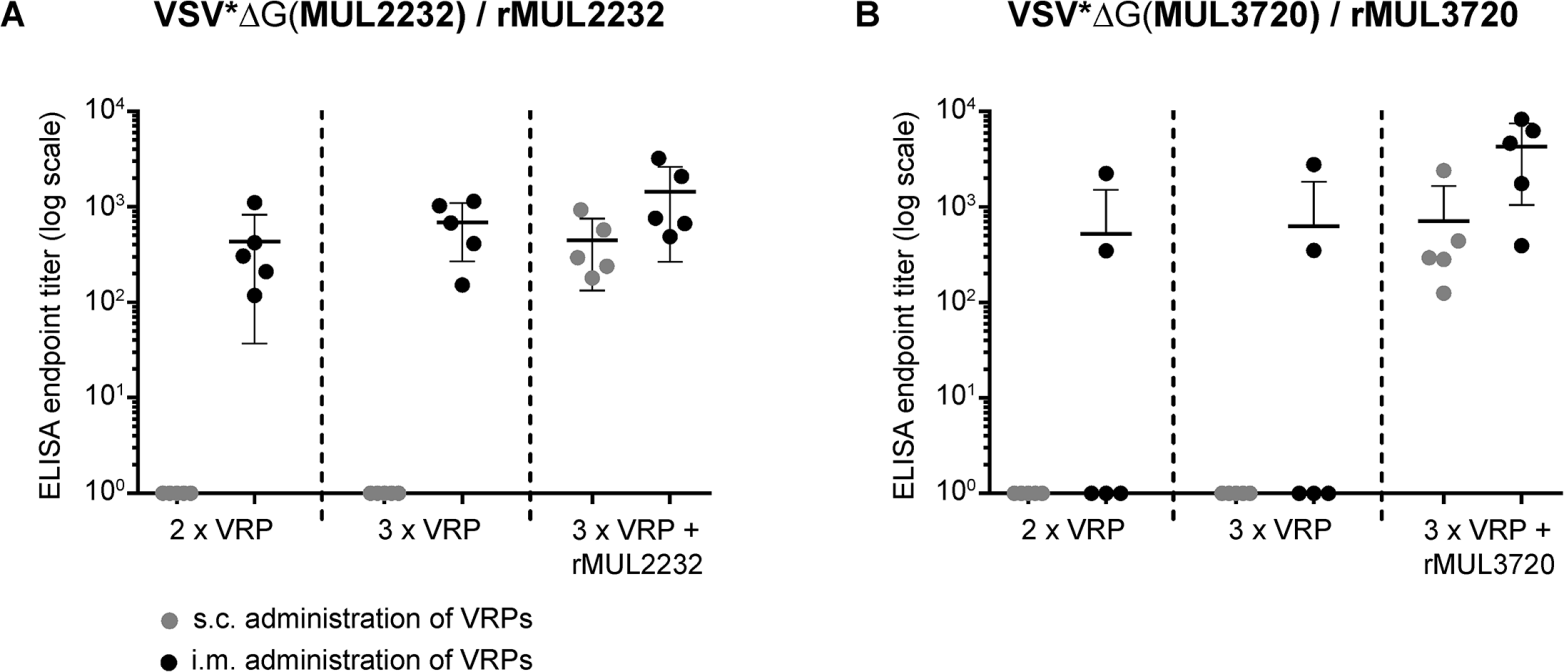
Comparison of immunization routes. Groups of five female BALB/c mice were sequentially immunized with VSV*ΔG(MUL2232) (A) or VSV*Δ(MUL3720) (B) via the sub-cutaneous (grey dots) or intra-muscular (black dots) route. Prior to every new immunization, blood was taken and serum analysed by ELISA on the corresponding recombinant proteins (rMUL2232 or rMUL3720). After the third immunization, all mice were boosted (s.c) with the VRP correspondent, non-adjuvanted recombinant protein. Endpoint total IgG titers were determined for individual animals in one single ELISA. Mean values (line) ± standard deviations are shown.

To characterize the immune responses elicited by the recombinant VRPs, we compared different immunization regimens in BALB/c mice. Humoral immune responses were assessed by ELISA on immobilized recombinant protein. Sub-cutaneous (s.c.) administration of 10^7^ VRPs did not lead to measureable antibody responses against the target antigens (Fig 2A and 2B). However, when VSV*ΔG(MUL2232) was given i.m., Immunoglobulin (Ig) G antibodies were elicited in all immunized animals. In contrast, only some mice produced antibodies following i.m. immunization with VSV*ΔG(MUL3720) (Fig 2A and 2B). Therefore induction of antibodies solely by i.m. immunization with VSV*ΔG(MUL3720) was no longer pursued in subsequent experiments. Independently of the route of administration, both VRPs primed the immune system, as demonstrated by the fast humoral immune response to an adjuvant-free booster immunization (s.c.) with 30 μg of the corresponding recombinant protein (Fig 2A and 2B). Six days after the booster injection, antibody titers were generally higher in mice primed i.m. than in mice primed s.c. (Fig 2A and 2B). Therefore, only the i.m. route was employed for VRP administration in subsequent experiments. In addition, a dose of 10^7^ VRPs per immunization was generally used.

**Fig 3:**
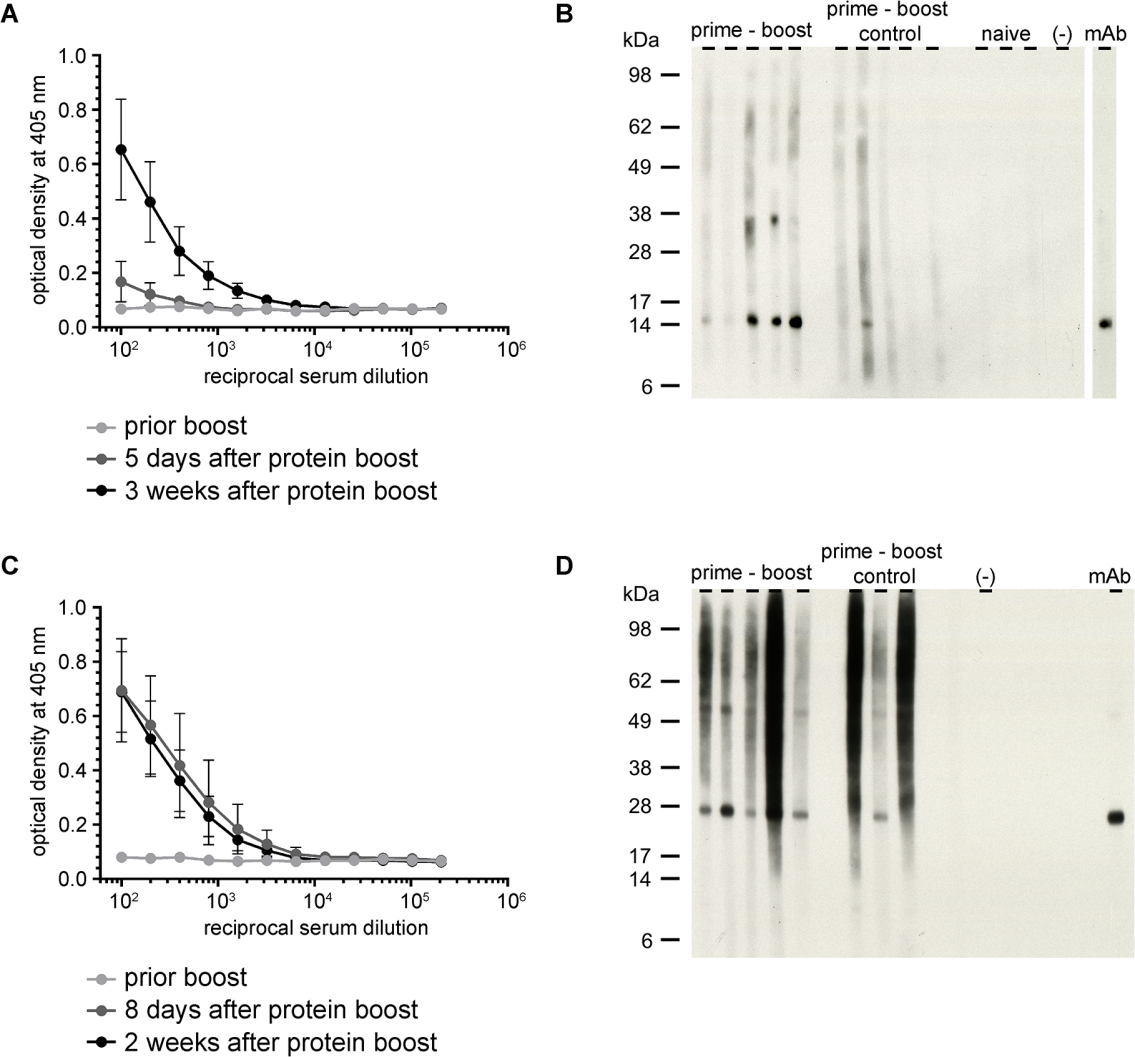
Induction of antigen-specific antibodies by a short prime-boost immunization protocol. Female BALB/c mice (n = 5) were immunized once with VSV*ΔG(MUL2232) or VSV*ΔG(MUL3720) and boosted with rMUL2232 or rMUL3720 three weeks later. Control animals (n = 5 and n = 3) were immunized with empty replicon prior to recombinant protein boost three weeks later. (A) Analysis of serially diluted immune sera by ELISA on immobilized rMUL2232. Mean values (dot) ± standard deviations are shown. (B) Western blotting analysis on *M*. *ulcerans* lysate using the indicated immune sera taken three weeks after protein boost. (C) Analysis of serially diluted immune sera by ELISA on immobilized rMUL3720. Mean values (dot) ± standard deviation are shown. (D) Western blotting analysis on *M*. *ulcerans* lysate using the indicated immune sera taken two weeks after protein boost.

In a next step, we assessed the potential of a shorter prime-boost immunization strategy by immunizing the animals only once i.m. with VSV*ΔG(MUL2232) or VSV*ΔG(MUL3720) and three weeks later with 30 μg of rMUL2232 or rMUL3720 via the subcutaneous route in the absence of adjuvant. Five days after the rMUL2232 boost, the ELISA IgG titers were only marginally higher than those observed prior to the boost, but increased further in the subsequent two weeks (Fig 3A). On the other hand, administration of rMUL3720 led to high antibody titers already eight days after the boost (Fig 3C). Importantly, the elicited antibodies were not only cross-reactive with the recombinant proteins produced in *E*. *coli* but also with the target proteins expressed by *M*. *ulcerans*, as demonstrated by Western blotting on *M*. *ulcerans* lysate (Fig 3B and 3D).

**Fig 4:**
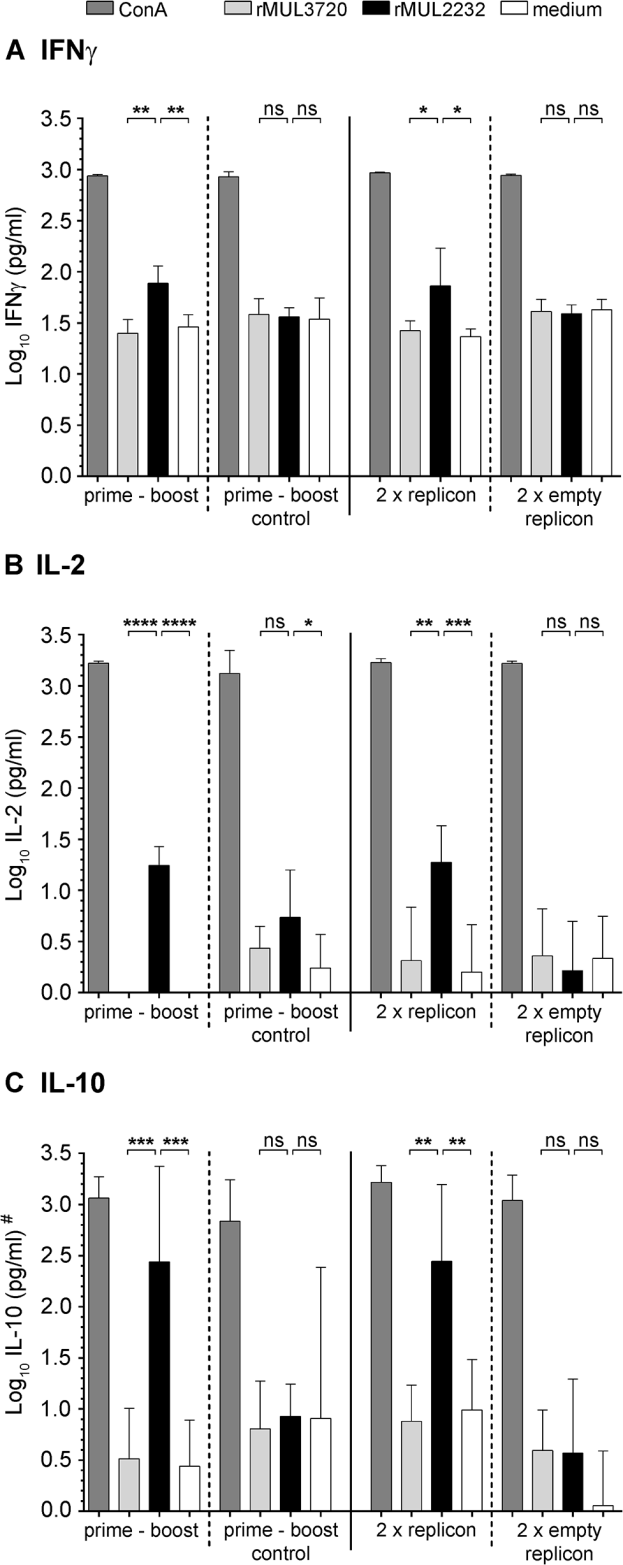
Analysis of cellular immune responses. Female BALB/c mice (n = 5) were immunized once with VSV*ΔG(MUL2232) (i.m.) and boosted with rMUL2232 (s.c., prime-boost) or were immunized with VSV*ΔG (i.m.) and boosted with rMUL2232 (s.c., prime-boost control). Alternatively, the animals were immunized twice with either VSV*ΔG(MUL2232) (2 x VRP) or the control vector VSV*ΔG (2 x empty VRP). Three weeks after the final immunization, mice were euthanized. Spleen cells were taken into culture and stimulated with either Concanavalin A (ConA), rMUL2232, rMUL3720 or left unstimulated (medium). Levels of secreted cytokines were determined by commercially available ELISA kits at 24 hours (IL-2, B) and 96 hours (IFNγ, A; IL-10, C) after stimulation. Mean values (bar) ± standard deviations for 5 mice tested are shown and expressed as log_10_ (pg/ml). * *p* ≤ 0.05; ** *p* ≤ 0.01; *** *p* ≤ 0.001; **** *p* ≤ 0.0001 (*p*–value adjusted for random effects in a linear mixed model). Ns equals non-significance. # Due to high non-specific rMUL3720 stimulation presumably caused by contaminants in the recombinant protein preparation, values were normalized to those of a non-immunized group of mice and z-values are depicted for all stimulations except ConA.

To evaluate whether this immunization regimen would also elicit antigen-specific cellular immune responses, antigen-specific cytokine secretion of spleen cells was studied *in vitro* after primary immunization of mice with VSV*ΔG(MUL2232) and a second immunization with either rMUL2232 or VSV*ΔG(MUL2232). Three weeks after the second immunization, spleen cells were stimulated with either rMUL2232 or the unrelated rMUL3720 as control. Stimulation with Concanavalin A (ConA) served as positive control for confirming the viability of the cultured spleen cells. The stimulated cell culture supernatants were analysed by ELISA for the production of the T_H_1 cytokine Interferon gamma (IFNγ) (Fig 4A), the pleiotropic [46] cytokine Interleukin 2 (IL-2) (Fig 4B) and the T_H_2 cytokine Interleukin 10 (IL-10) [47] (Fig 4C). Overall, no marked difference in terms of cytokine production was observed between the two immunization schedules. In both cases all three cytokines were produced in significantly higher amounts when cultured spleen cells were stimulated with the corresponding rMUL2232 antigen. In contrast, stimulation with the unrelated antigen rMUL3720 or mock stimulation had no significant effect (Fig 4). The most pronounced response was found for IL-10 (Fig 4C), indicating a slight polarization towards a T_H_2 type response. The same experimental setup with VSV*ΔG(MUL3720) was not successful due to technical problems.

### Vaccination has no marked inhibitory effect on bacterial proliferation in an experimental BU mouse model

**Fig 5:**
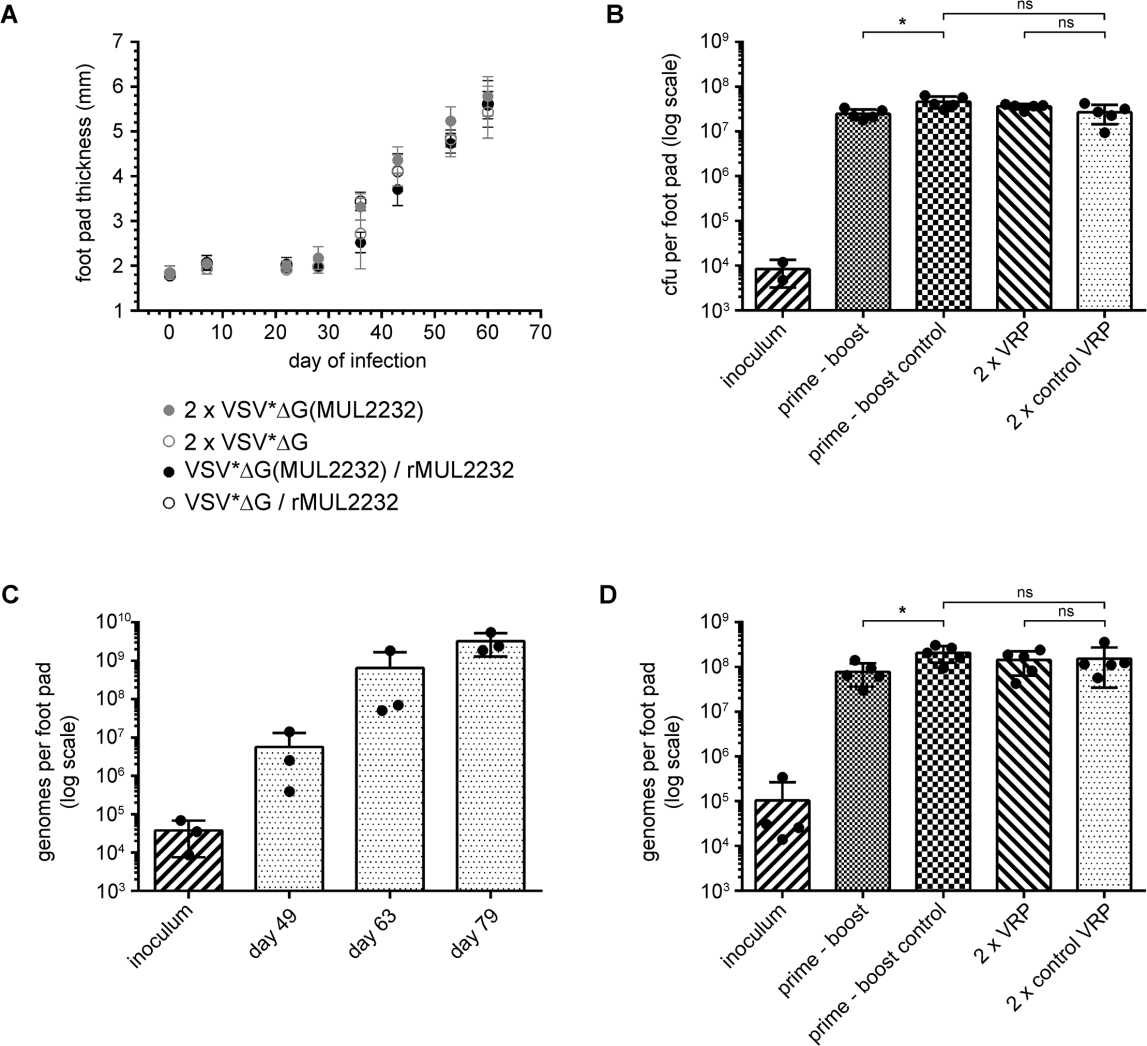
Infection of immunized mice with *M*. *ulcerans*. Groups of six immunized, female BALB/c mice were infected by s.c. injection of *M*. *ulcerans* suspension (30 μl) into the left hind foot pad. (A) Development of the infection was followed by weekly measurements of the foot pad thickness with a caliper. The mean foot pad thickness (dot) ± standard deviation is shown for each animal group. (B) At day 60 after infection, mice were sacrificed and the number of *M*. *ulcerans* bacilli in foot pads determined by classical CFU plating. (C) Quantification of *M*. *ulcerans* in foot pad lysates by IS2404 specific qPCR. Genome equivalents are shown. (D) Determination of *M*. *ulcerans* genome equivalents in immunized and infected mice. * *p* ≤ 0.05 (Mann-Whitney test).

In a last step, we explored the protective efficacy of the VRP-based immunization in an experimental *M*. *ulcerans* infection model. Groups of six BALB/c mice were immunized according to the VSV*ΔG(MUL2232)/rMUL2232 prime-boost regimen or two times i.m. with VSV*ΔG(MUL2232). Respective control groups were immunized either with the control VRP VSV*ΔG and an rMUL2232 boost (prime-boost control) or twice with VSV*ΔG (Fig 5). Three weeks after the last immunization, the left hind foot pad of the mice was infected with 8.4 x 10^3^ *M*. *ulcerans* bacilli. The slowly progressing infection was followed by weekly measurements of the thickness of the infected foot pads with a caliper (Fig 5A). To determine the bacterial multiplication, mice were euthanized at day 60 after infection and foot pads either processed for histopathological analysis or lysed for enumeration of *M*. *ulcerans* by standard CFU plating. Quantification was additionally performed by an adapted qPCR method, suitable for the detection of bacterial proliferation in mouse foot pads over the course of the infection (Fig 5C).

No differences in the kinetics of footpad swelling were observed between immunized mice and the respective control animals (Fig 5A). However, 60 days post infection the number of CFU per foot pad was slightly but significantly lower in animals immunized with the VRP prime-protein boost regimen as compared to the prime-boost control immunized animals (Fig 5B). Quantification of *M*. *ulcerans* DNA in the footpads by insertion sequence (IS) 2404 specific qPCR yielded similar results, i.e. a slight but significant reduction in bacterial multiplication caused by the VRP prime-protein boost immunization, but not by two subsequent immunization with VRPs (Fig 5B and 5D). The same experiment with MUL3720 VRPs did not result in any difference between immunized and control groups (S1 Fig).

**Fig 6:**
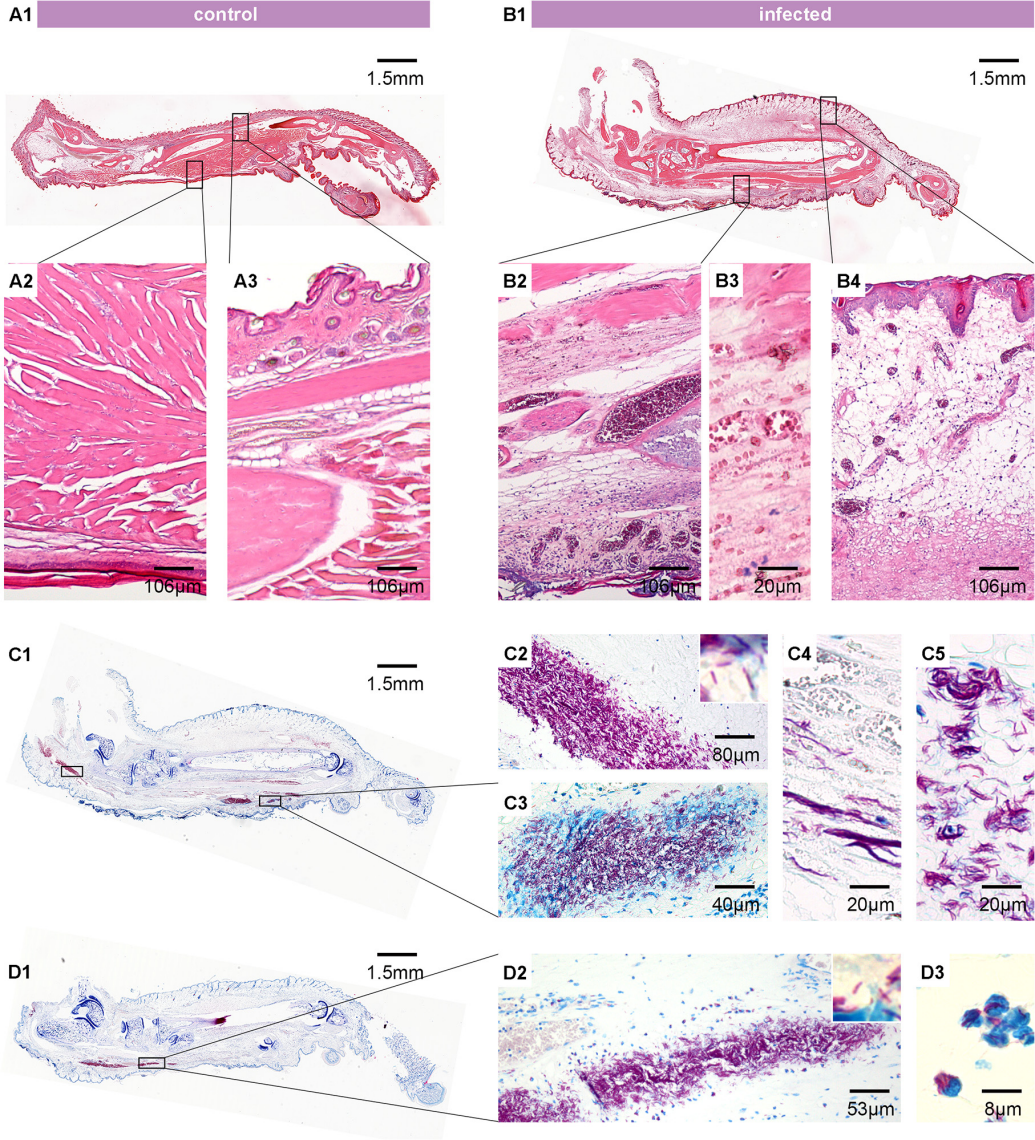
Histopathological analysis of mouse foot pads following infection with *M*. *ulcerans*. Histological sections of foot pads from *M*. *ulcerans*-infected mice were stained with Haematoxylin/Eosin (A1-A3, B1-B4) or Ziehl-Neelsen/Methylene blue (ZN) (C1-C5, D1-D3). The right, non-infected foot pad showed no swelling (A1), intact muscle tissue (A2) and absence of oedema in the upper part of the foot (A3). As opposed to this, the left foot of a mouse immunized with VSV*ΔG/rMUL2232 (prime-boost control) was strongly swollen 60 days post infection (B1). Cellular infiltration and beginning necrosis of the tissue was explicitly strong at the base of the foot pad, where bacteria were injected (B2) and muscle fibres started to become destroyed (B3). The upper part of the foot is highly oedematous (B4). Staining of the same foot pad for AFB revealed large amounts of bacteria in close contact to infiltrating immune cells at the base of the foot (C3). Appearance of AFB in the destroyed muscle tissue (C4). Big clumps of bacteria were also found towards the ankle of the foot (C2) and in the oedematous tissue in the upper half of the foot pad (C5). The foot pad of a mouse immunized with VSV*ΔG(MUL2232)/rMUL2232 (prime-boost) contained large amounts of AFB (D1) which were less spread in the whole foot pad. Intracellular AFB were frequently observed (D3).

A histopathological analysis of representative foot pads confirmed the findings of a slight reduction of bacterial burden in the VSV prime-protein boost immunized animals as compared to the prime-boost control animals. The non-infected right foot pads served as control and appeared completely normal (Fig 6A1) with intact muscle tissue (Fig 6A2) and no apparent oedematous changes (Fig 6A3). In comparison, the infected left foot pads of control mice showed typical histopathological signs of BU with strong oedema (Fig 6B1 and 6B4), necrotic sole of foot (Fig 6B2), and inflammatory infiltration and extensive haemorrhages all over the foot pad (Fig 6B3). Ziehl-Neelsen/Methylene blue (ZN) staining revealed large clumps of acid fast bacilli (AFB) (Fig 6C1), not only located where they were initially injected, but also more towards the heel of the foot (Fig 6C2) and in the oedematous tissue in the upper part of the foot pad (Fig 6C5). AFB were associated with remains of infiltrating immune cells (Fig 6C3) and were also found as fibrous structures in completely necrotic tissue (Fig 6C4). In animals that received a VRP prime-protein boost immunization, a trend towards a reduction in the number of AFB clusters was observed (Fig 6D1). Furthermore, AFB appeared to be more often in close contact with infiltrating cells or were found intracellular (Fig 6D3).

## Discussion

Despite substantial control efforts and improvements in treatment and diagnosis, the socioeconomic impact of BU on affected communities remains high. Long treatment regimens with daily i.m. injections in rural settings of West Africa, late reporting to health facilities, scarring and resulting permanent disabilities create high morbidity that could be prevented by a vaccine against *M*. *ulcerans*. Although no such vaccine is available at the moment, reports on self-healing in patients with early stages of the disease [48,49] as well as sero-epidemiological studies in BU-endemic countries [32,50] indicate that the human organism is in principle capable of inducing a protective immune responses against BU. Furthermore, protein subunit vaccination approaches have demonstrated partial protection in a BU mouse infection model [21,22,51].

In the present study, we used a viral replicon particle system for delivering *M*. *ulcerans* protein antigens to the immune system. Immunization with a high number of VRPs induced *M*. *ulcerans* specific antibody responses with i.m. application being superior to s.c. application. Our VRP prime-recombinant protein boost regimen not only induced a humoral but also a cellular immune response. This is in line with previous work showing that VRPs are able to trigger humoral as well as cellular immune responses [52].

The bacterial proteins MUL2232 and MUL3720 were chosen as vaccine antigens because previous work suggested that these antigens are not only highly immunogenic but also associated with the outer surface of *M*. *ulcerans* [32,34,35]. Therefore, the strong humoral immune response observed following immunization of mice was expected to allow antibody-mediated opsonization of the bacteria with consequent enhanced phagocytosis, complement activation, or antibody-dependent cellular cytotoxicity. While we did not observe vaccination-induced reductions in foot pad swelling in our murine *M*. *ulcerans* infection model, assessment of the bacterial load by qPCR as well as CFU plating revealed a slight, but significant reduction in bacterial multiplication in VRP prime-protein boost immunized mice. While measurement of foot pad swelling over time is a parameter that can be followed without euthanizing mice [53,54], our results illustrate that foot pad swelling not necessarily reflects the extent of bacterial proliferation.

Despite a slight inhibitory effect on bacterial proliferation, immunization with only one target antigen in a VRP prime-protein boost regimen was not sufficient to confer full protection against the experimental infection. Several factors, like the choice or number of antigens included into the VRPs, could have let to this negative result. Since it is not known, which immune effector functions are relevant for protection against *M*. *ulcerans* disease [15], it is not clear whether lack of a strong T_H_1-polarisation is of major relevance for the failure to achieve a strong protective efficacy with the immunization regimens tested. One of the advantages of the replicon system lies in the ability of VSV to tolerate incorporation of long stretches of foreign DNA into its genome [25,26]. As a modular system, it offers the possibility to design a multivalent subunit vaccine by combining several *M*. *ulcerans* proteins in one replicon. Furthermore the versatility of the system allows to engineer the location of the expressed protein in the infected cell, or to target specific immune cells by including genes for co-stimulatory molecules or receptors to be expressed on the surface of the infected cells [25]. Here we have demonstrated that RNA replicon particles are a very good delivery system for mycobacterial antigens, which is in particular encouraging future development of VRP-based multivalent subunit vaccines.

## Supporting Information

S1 Fig Infection of immunized mice with *M*. *ulcerans* (MUL3720 immunizations). Groups of six immunized, female BALB/c mice were infected into the left hind foot pad with 30 μl of *M*. *ulcerans* suspension (s.c.). (A) Development of the infection was followed by weekly measures of the foot pad thickness with a caliper. Depicted is the mean foot pad thickness (dot) ± standard deviation of the individual differently immunized groups. (B) At day 60 after infection, mice were sacrificed and the number of *M*. *ulcerans* bacilli in foot pads determined by classical CFU plating. (C) Determination of *M*. *ulcerans* genome equivalents in immunized and infected mice.
(PDF)
