# Supplementary material for: Use of Recombinant Virus Replicon Particles for Vaccination against Mycobacterium ulcerans Disease
Source: PLoS Negl Trop Dis. 2015 Aug 14;9(8):e0004011. doi: 10.1371/journal.pntd.0004011 (PMC4537091; doi:10.1371/journal.pntd.0004011)
Supplement: S1 Fig — Groups of six immunized, female BALB/c mice were infected into the left hind foot pad with 30 μl of M. ulcerans suspension (s.c.). (A) Development of the infection was followed by weekly measures of the foot pad thickness with a caliper. Depicted is the mean foot pad thickness (dot) ± standard deviation of the individual differently immunized groups. (B) At day 60 after infection, mice were sacrificed and the number of M. ulcerans bacilli in foot pads determined by classical CFU plating. (C) Determination of M. ulcerans genome equivalents in immunized and infected mice. (PDF) [file pntd.0004011.s001.pdf]

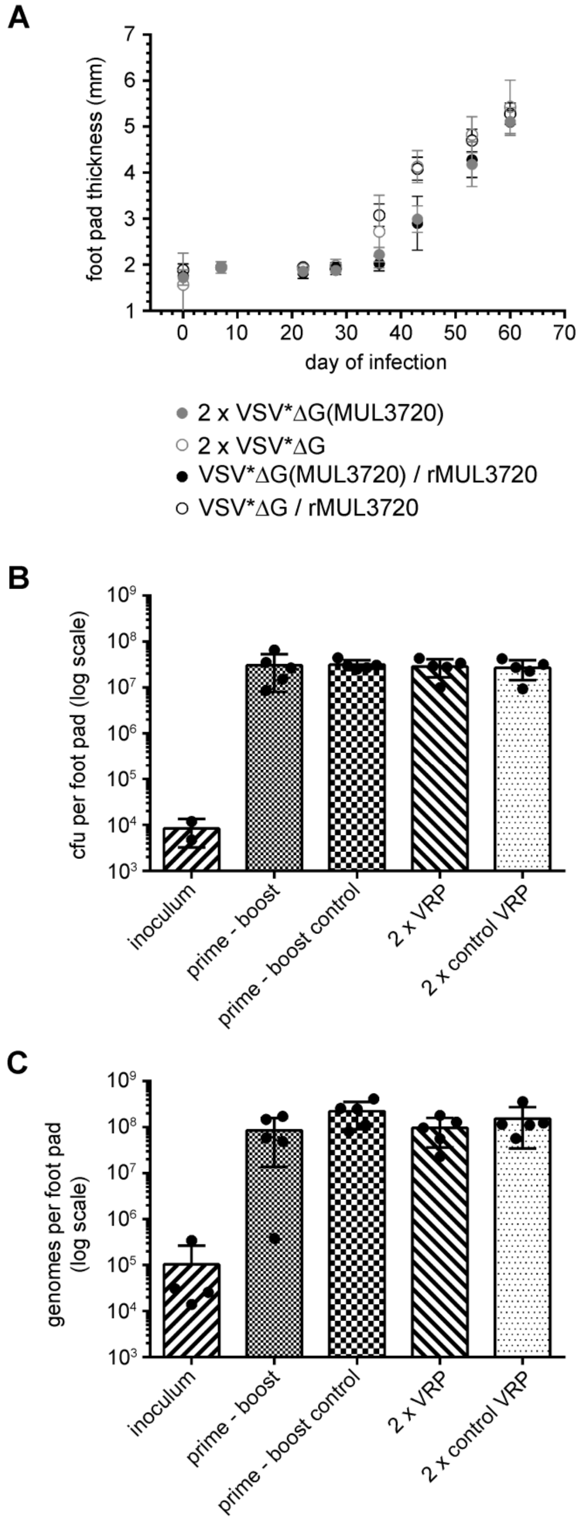

Supplementary Figure 1: Infection of immunized mice with *M. ulcerans* (MUL3720 immunizations).

Groups of six immunized, female BALB/c mice were infected into the left hind foot pad with 30 µl of *M. ulcerans* suspension (s.c.). (A) Development of the infection was followed by weekly measures of the foot pad thickness with a caliper. Depicted is the mean foot pad thickness (dot) ± standard deviation of the individual differently immunized groups. (B) At day 60 after infection, mice were sacrificed and the number of *M. ulcerans* bacilli in foot pads determined by classical CFU plating. (C) Determination of *M. ulcerans* genome equivalents in immunized and infected mice.
